# Biomaterials, Composites, and Nanotechnology: The Amazonian Potentialities

**DOI:** 10.3390/biotech15040091

**Published:** 2026-10-11

**Authors:** Eduarda Lemos, Monize Bürck, Marcelo Assis, Anna Rafaela Cavalcante Braga

**Affiliations:** 1 Postgraduation Program in Nutrition, Universidade Federal de São Paulo (UNIFESP), Botucatu Street, 740, Vila Clementino, São Paulo 04023-062, SP, Brazil; eduarda.lemos@unifesp.br; 2 Nutrition and Food Service Research Center, Universidade Federal de São Paulo (UNIFESP), Silva Jardim Street, 136, Vila Mathias, Santos 11015-020, SP, Brazil; monize.burck@unifesp.br; 3 Institute for Advanced Marine Studies (IEMar), Universidade Estadual de São Paulo (UNESP), São Vicente 11350-011, SP, Brazil; marcelostassis@gmail.com; 4 Department of Biosciences, Universidade Federal de São Paulo (UNIFESP), Silva Jardim Street, 136, Vila Mathias, Santos 11015-020, SP, Brazil; 5 Department of Chemical Engineering, Universidade Federal de São Paulo (UNIFESP), São Nicolau Street, 210, Centro, Diadema 09972-270, SP, Brazil

**Keywords:** biodiversity, bioresources, biomass valorization, encapsulation, bioactive compounds, Amazon, biomaterials, biorefinery, circular economy, nanotechnology

## Abstract

Pan-Amazonian biodiversity represents an unexplored source of high-value compounds and renewable raw materials, particularly from the many plant species of the Amazon Forest. Although the legal framework for Amazonian resource exploitation is grounded in sustainable management systems, generating a significant amount of plant residues remains unavoidable, creating opportunities to valorize them within circular economy frameworks. Amazonian biomass and its byproducts can provide both structural biopolymers and reinforcing materials (i.e., cellulose, starch, proteins, and fibers) and bioactive compounds (i.e., phenolic compounds, pigments, and essential oils). These compounds are valuable because of their antioxidant, antimicrobial, and anti-inflammatory properties; however, their application is often limited by chemical instability, low bioavailability, and susceptibility to processing and storage conditions. Innovative composites and micro- and nano-materials address these challenges, enabling applications in the food, cosmetic, biotechnology, and pharmaceutical industries. This review critically examines current approaches, emerging trends, and knowledge gaps in extracting, processing, and transforming Amazon resources into micro- or nano-structured biomaterials, demonstrating the relationships among their composition, material properties, the biological effects of bioactive compounds, and applications.

## 1. Introduction

The Amazon, a biome with approximately 60% of its area within Brazilian territory, is one of the world’s major reservoirs of biodiversity, encompassing extraordinary diversity of plant, animal, and microbial species, along with remarkable genetic and chemical diversity [1,2]. This complex biological diversity extends beyond its ecological importance and represents a valuable source of natural resources with potential applications in the food industry [3], pharmaceuticals [4], cosmetics [5], and biotechnology [6].

Characterization studies of fruit species from the region, for example, have identified phenolic compounds, flavonoids, carotenoids, and other metabolites associated with antioxidant and anti-inflammatory properties [5,6]. In parallel, various Amazonian plant matrices contain structural and nutritional components, such as lipids and fibers, which can be recovered and valorized for higher-value applications [7]. In this context, Amazonian biodiversity can be regarded not only as an environmental heritage but also as an important reservoir of raw materials and molecules that can support innovative strategies and bioproduct development.

The sustainable use of these resources has contributed to the development of various value chains associated with the Amazonian bioeconomy. Fruits, seeds, leaves, peels, oils, and other fractions derived from native species, such as açaí (*Euterpe oleracea*) [8], buriti (*Mauritia flexuosa*) [9], cupuaçu (*Theobroma grandiflorum*) [10], castanha-do-Brasil (*Bertholletia excelsa*) [11], bacaba (*Oenocarpus distichus*) [12], pracaxi (*Pentaclethra macroloba*) [13], and cultivated species, such as malva (*Malva sylvestris*), cúrcuma (*Curcuma longa*), and mandioca (*Manihot esculenta*), have been investigated as sources of compounds of interest due to their chemical composition and functional properties, which favor applications beyond their traditional uses.

Recent literature has also highlighted the potential of more sustainable extraction and processing technologies for these resources, enabling a shift away from models focused on obtaining a single product of interest and promoting biomass fractionation for the recovery of both high- and low-value compounds, thereby integrating the concepts of biodiversity, technological innovation, and the bioeconomy [14].

In this context, recent studies have demonstrated that Amazonian biomass and co-products can provide both structural components and bioactive compounds, and different processing strategies have therefore been employed to recover and transform these fractions. For açaí and cupuaçu, for example, researchers have investigated processing by-products and co-products as sources of various compounds of interest for a wide range of applications [15,16,17,18]. These studies show that valorizing residual biomass is an established research field and highlight the potential to use different components from the same raw material for distinct applications.

This perspective is directly related to the biorefinery concept, in which biomass is regarded as an integrated source of different components that can be separated, transformed, and directed toward different products according to their characteristics and added value [19,20]. Rather than restricting raw material use to producing a single product, fractionation and processing strategies can enable sequential recovery of different constituents. Within this framework, a stream generated at a given processing stage can serve as feedstock for a subsequent step, establishing cascade valorization systems and reducing losses throughout the production chain [21].

Beyond recovering compounds of interest, structural fractions from these biomasses show considerable potential for developing biomaterials and composites. Lignocellulosic fibers, cellulose, starch, and other biopolymers can serve as matrices, fillers, or reinforcing agents, contributing to materials with enhanced mechanical, thermal, barrier, and functional properties. This shows that co-products and residual biomasses can move beyond their conventional classification as low-value by-products and instead serve as raw materials for developing high-value materials [22,23,24].

In parallel, the bioactive compounds in these biomasses have properties of interest for food, pharmaceutical, cosmetic, and biotechnological applications, particularly because of their antioxidant [25] and anti-inflammatory activities [26]. However, their chemical instability, low bioavailability, and susceptibility to processing and storage conditions, as well as to factors such as light, temperature, oxygen, and pH, may limit their direct application [27,28]. In this context, micro- and nano-structuring strategies can broaden their potential applications by enabling incorporation into systems designed to improve stability, protection, and release, while also enhancing interactions with different matrices [29,30].

The combination of the chemical diversity of Amazonian biomass with the possibilities offered by micro- and nano-structuring technologies therefore creates opportunities to develop multifunctional biomaterials and composites. Accordingly, this review critically examines current approaches, emerging trends, and knowledge gaps related to the extraction, processing, and transformation of resources from the Amazon into biomaterials, composites, and micro- and nano-structured matrices, with particular emphasis on the relationships among their chemical composition, material properties, biological effects of bioactive compounds, and potential applications. The literature review also identifies the main biomasses and fractions investigated, the strategies used for their valorization, and the material platforms developed, contributing to a broader understanding of opportunities to integrate biodiversity, biorefinery concepts, circular economy principles, and the development of composites and other bio-based materials.

## 2. Methodology

**Figure 1:**
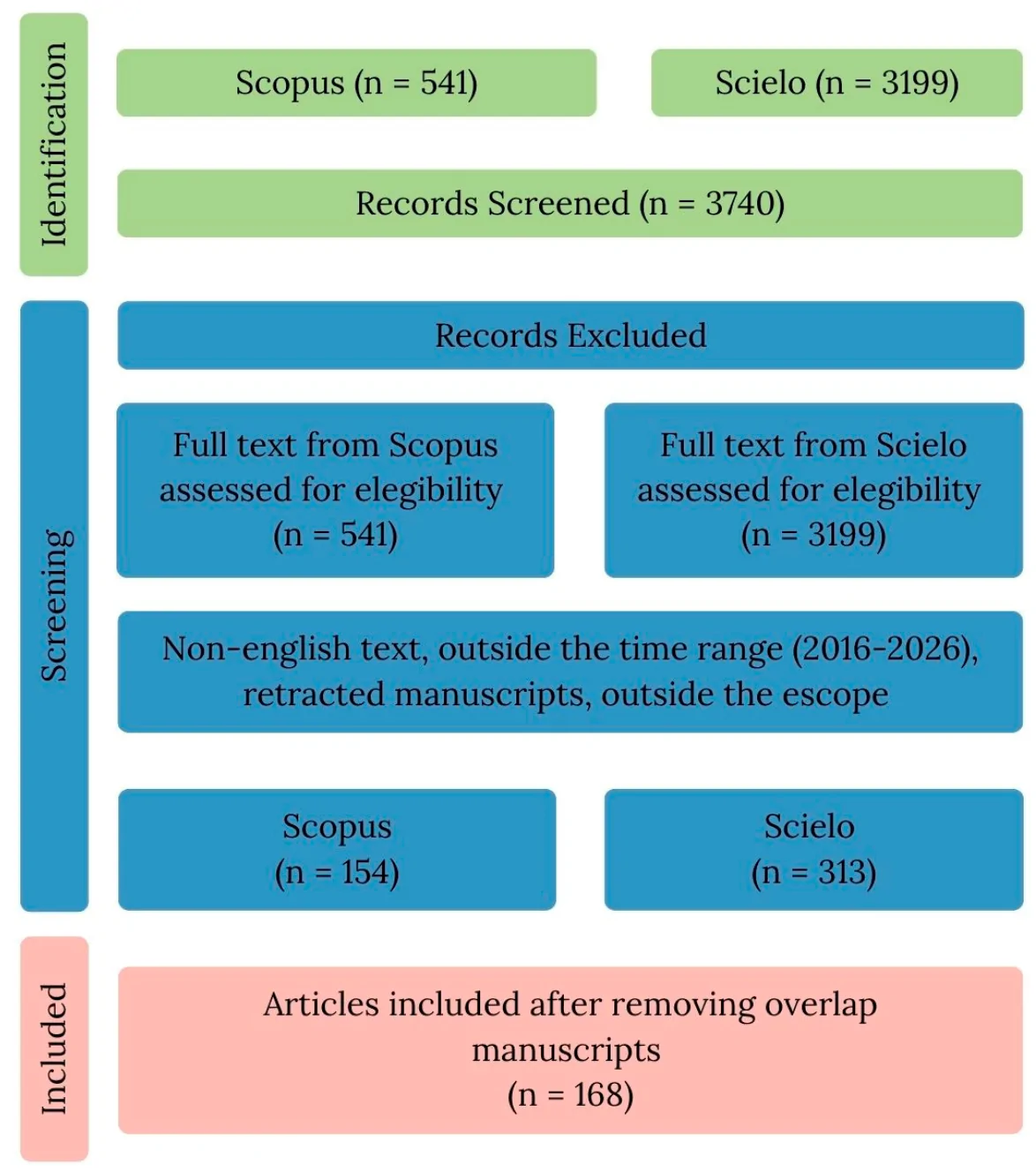
PRISMA flow diagram for the systematic review detailing the database searches, the number of documents screened, and the full texts assessed for eligibility.

A structured literature search was conducted in the Scopus and SciELO databases to identify studies on valorizing Amazonian biological resources for developing biomaterials, composites, and micro- and nano-structured systems. The search covered studies published between 2016 and 2026 and organized them around the review’s main thematic domains, including plant-derived structural components, bioactive compounds and natural extracts, micro- and nano-technology approaches, technological applications, and biomass valorization within sustainability and circular economy frameworks. The initial search yielded 3199 records in SciELO^®^ and 541 documents in Scopus^®^. After applying predefined eligibility criteria, we retained 168 scientific articles for detailed analysis (Figure 1). The search was conducted in July 2026. Some articles published prior to 2016 were added to enhance the discussion and provide a broader overview of the study.

The inclusion criteria for academic literature were restricted to original research articles published between 2016 and 2026 that investigated Amazonian resources for new biomaterials to produce composites and apply in nanotechnology studies. Non-English documents, review papers, and studies that discussed potential applications without experimental formulation data were excluded.

Amazon-related descriptors, applied Amazon-related descriptors and domain-specific terms to titles, abstracts, and keywords, and evaluated results for original research articles directly addressing Amazonian biological resources, their derived fractions, or processing residues and co-products. A complementary bibliometric analysis characterized the research landscape and identified thematic relationships and temporal trends associated with Amazonian resources and material development. Bibliographic records were retrieved from both databases using the following search strategy: TITLE-ABS-KEY (“Amazon” OR “Amazonian” OR “Amazon rainforest”) AND TITLE-ABS-KEY (“microstructure” OR “nanostructures” OR “biomaterials” OR “composites”).

**Figure 2:**
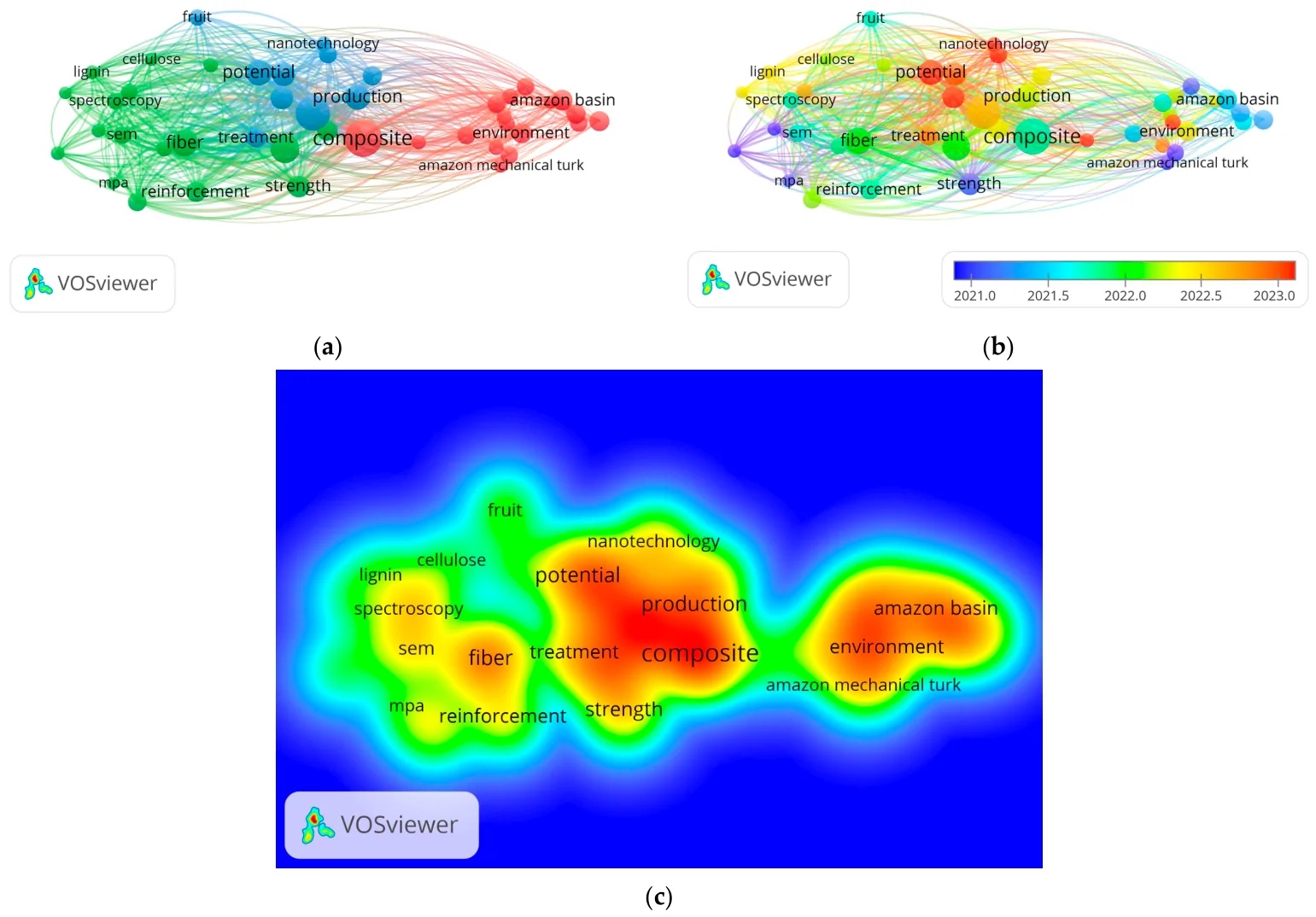
Bibliometric map from bibliographic data across article titles, abstracts, and keywords in the Scopus^®^ and SciELO^®^ databases using the keywords most frequently cited in the manuscripts evaluated: (**a**) maps for clusters; (**b**) maps for temporal distribution; and (**c**) density visualization.

The search returned records, which we exported and analyzed using VOSviewer 1.6.21. Term co-occurrence analysis across titles, abstracts, and keywords was used to construct network and temporal overlay visualizations, enabling the identification of thematic clusters and their distribution across the analyzed period (Figure 2).

## 3. Literature Overview

The VOSviewer network visualization (Figure 2a) revealed three main thematic clusters, suggesting the literature is structured around environmental, materials science, and applied value-chain perspectives related to Amazonian resources [31,32,33,34].

The red cluster is centered on the Amazon and its environmental context. Keywords such as Amazon, Amazon rainforest, Amazonia, Brazilian Amazon, Peru, and South America locate the research geographically, while climate change, deforestation, precipitation, environment, and expansion indicate a strong focus on ecological transformation and regional environmental dynamics [35,36,37,38,39,40]. The presence of Amazon Mechanical Turk may reflect studies that use this platform for surveys, perception studies, or data collection, rather than a direct connection to the Amazon biome. The terms composite, composite analysis, and composite index suggest integrative analytical approaches to assess environmental conditions, land-use changes, or socioeconomic and ecological indicators.

The green cluster represents the material characterization of Amazon-derived natural fibers and lignocellulosic resources. Terms such as cellulose, lignin, natural fiber, reinforcement, mechanical properties, strength, and morphology indicate interest in using these materials as reinforcements in composites. Recurring analytical techniques, such as FTIR, SEM, electron microscopy, spectroscopy, and X-ray diffraction, indicate that this body of literature focuses on chemical composition, microstructure, crystallinity, and mechanical performance. Overall, this cluster highlights Amazonian biomass’s potential as a sustainable source of advanced materials.

The blue cluster focuses on extracting, processing, characterizing, and applying plant- and fruit-derived products. Keywords such as extraction, characterization, production, treatment, stability, and application point to research aimed at converting biological resources into usable products. The inclusion of nanotechnology and potential suggests an emerging interest in high-value applications, including nano-enabled materials, bioactive compounds, and improved formulations. This cluster therefore emphasizes the technological and economic valorization of Amazonian biodiversity [41,42].

The clusters show an interdisciplinary field in which environmental issues affecting the Amazon coexist with research on characterizing and sustainably exploiting its biological resources. They also suggest a potential research pathway linking conservation and responsible resource management with the development of natural-fiber composites, plant-based extracts, and innovative bio-based applications.

The correlation between material sources and characterization parameters further suggests that optimizing mechanical and physicochemical properties remains a key issue in defining potential applications and, consequently, the technological valorization of Amazonian biomass [35,43,44,45]. When the temporal overlay visualization is analyzed (Figure 2b), these thematic relationships indicate a progressive transition across research fields, and sustainability appears to function not only as an independent research topic but also as a conceptual link between conserving Amazonian resources and transforming them into higher-value materials [46,47,48].

Recent trends corroborate this scenario, indicating a broadening research scope. Terms such as biomaterials, antioxidant activity, and particle size appear in more recent publications, suggesting growing interest in applications beyond the conventional structural use of biomass. More specifically, the emergence of antioxidant activity may indicate growing attention to the functional and biological properties of Amazonian resources, complementing the established literature focused primarily on fibers and mechanical reinforcement [49,50].

The network and temporal evolution analyses indicate that research involving Amazonian resources is progressing toward greater integration among biodiversity, sustainability, materials science, and functional applications. However, the predominance and high connectivity of terms related to fibers, cellulose, composites, and mechanical properties demonstrate that structural applications remain considerably more established in the literature.

## 4. Amazonian Bioresources for Composite Materials Development

The compositional diversity of Amazonian biomass allows the same raw material to provide both structural components, such as fibers, cellulose, starch, and other polysaccharides, and bioactive compounds. This complementarity supports biorefinery exploitation by fractionating and valorizing different biomass components to obtain higher-value products [51,52]. Some reviews addressed Amazonian bioresources and their importance, but they were published before 2016 [23,53,54,55]. Other review works were more recently published but with a more specific focus, such as bioeconomy [55,56], antioxidant properties [57], natural fibers and composites [58], and Amazonian fruits [59].

Sequential strategies in which bioactive compounds are recovered before valorizing the remaining structural fractions show that these two resource classes can be integrated within the same valorization chain [48], as summarized in Figure 2. Therefore, assessing structural and bioactive components together is essential to understand Amazonian biomass’s potential as a raw material for integrated processes that maximize resource utilization.

### 4.1. Plant-Derived Biopolymers and Structural Components

**Figure 3:**
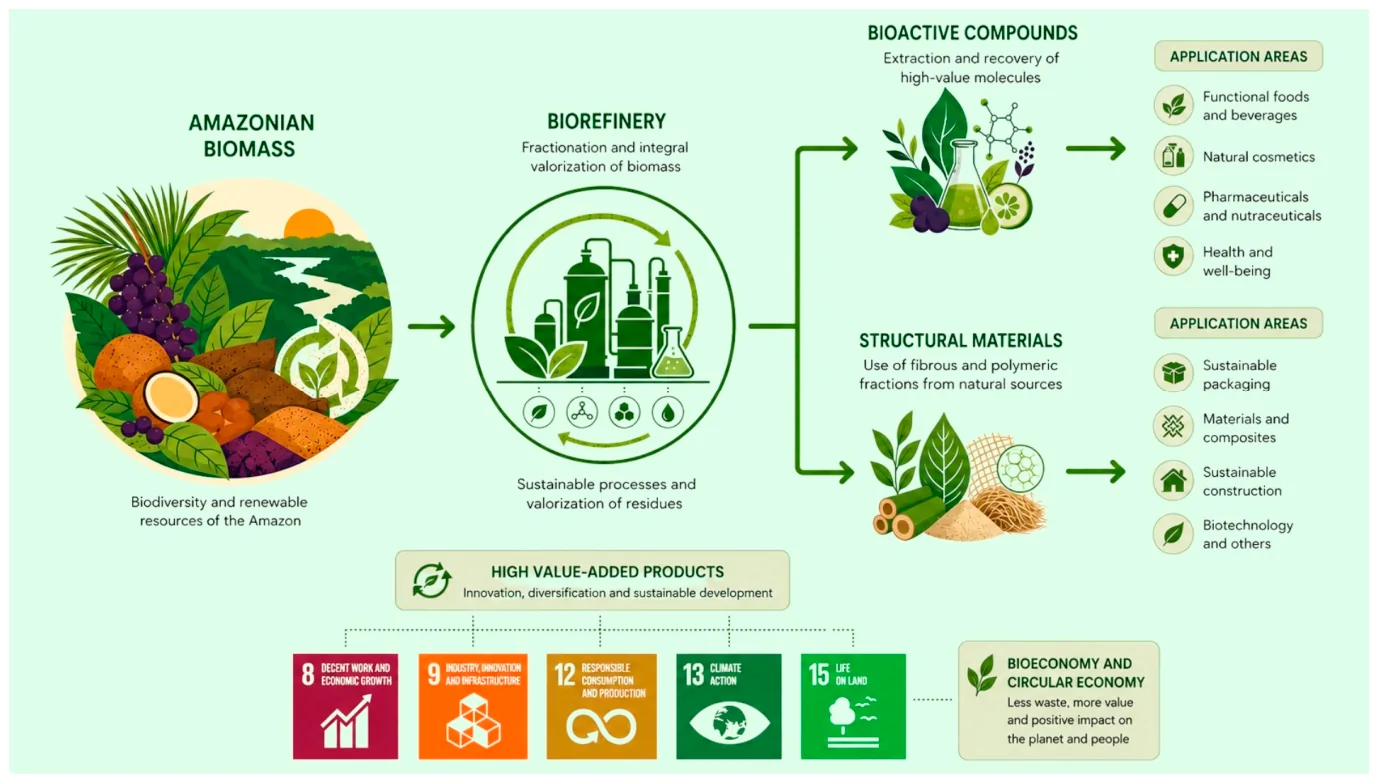
Schematic representation of the biorefinery-based valorization of Amazonian biomass.

Amazonian plant resources provide diverse structural components with potential for developing bio-based materials. Among the selected studies, lignocellulosic fibers, cellulose, nanocellulose, and starch stand out, along with less extensively explored polysaccharides, such as pectin [45,52,60]. Researchers have used these components for different technological purposes, from reinforcing polymeric matrices to developing films, bioplastics, and micro- and nano-structured materials. As summarized in Table 1, their applications are directly related to the raw materials’ characteristics and the modifications induced during processing. The schematic illustration in Figure 3 shows the importance of biorefinery-based valorization of Amazonian biomass.

Lignocellulosic fibers are primarily explored as reinforcing agents in composites, whereas cellulose isolation and nano-structuring broaden their potential applications, particularly by modulating structural, mechanical, and barrier properties [61,62]. In contrast, starch is predominantly used as a polymeric matrix, particularly to produce biodegradable films and packaging materials [61,63]. Although less frequently represented among the selected studies, pectin and other polysaccharides broaden the diversity of structural components that can be recovered from Amazonian biomass and residues and used for different technological applications.

The predominance of lignocellulosic fibers, cellulose, and starch among the structural components investigated may be attributed to the versatility of these fractions in performing different functions within materials. For fibers, their structure allows direct use as reinforcing agents, although performance depends on their interaction with the matrix. Ferreira et al. [60], for example, observed 48–72% increases in the elastic modulus of PBAT depending on the Amazonian fiber incorporated, identified fiber content as the main factor determining the tensile strength of curauá biocomposites, and showed that alkaline treatment also influences fiber–matrix adhesion [50]. The cellulosic fraction allows for even greater structural modification. Mathias et al. [62] obtained cellulose nanocrystals from jute and mallow fibers, with crystallinity increasing from approximately 60% to 85%, whereas Braga et al. [63] demonstrated that increasing the degree of nano-fibrillation of cellulose derived from açaí mesocarp increased crystallinity from 55% to 63% and reduced the water vapor permeability of the films from 62.51 to 36.75 g·mm·kPa^−1^·day^−1^·m^−2^. These findings help explain the technological interest in lignocellulosic materials. Beyond their direct use as reinforcing agents, their fractions can be isolated and processed at different scales to tailor specific material properties.

Starch offers a distinct advantage, as its ability to form a continuous polymeric phase enables its use as a matrix for films and biodegradable materials. This matrix can also incorporate other Amazonian components to modify its properties. Romani et al. [64] incorporated açaí seed flour into starch-based films and observed improved dispersion at a 5% incorporation level, along with the introduction of antioxidant activity, whereas Oliveira et al. [65] found that the addition of buriti oil to thermoplastic starch biocomposites containing ucuuba fibers reduced water absorption and improved flow during processing and fiber adhesion. Thus, the recurring presence of these biopolymers in the literature appears related not only to their availability in Amazonian biomass but also to their versatility as reinforcing agents, matrices, or raw materials for developing more highly processed structures, allowing their properties to be tailored to the intended application.

In contrast, pectin and other polysaccharides remain less extensively explored as structural components. The recovery of rhamnogalacturonan I-enriched pectin from Amazonian cocoa husks with a purity of 91.05% [66] and the characterization of a highly crystalline mannan from açaí seeds [67] demonstrate that other fractions can be recovered from these biomasses, although their transformation into materials has been less extensively demonstrated. In this context, the selected studies highlight different approaches to the technological valorization of Amazonian structural components. While fibers, cellulose, and starch are already being incorporated into materials with well-defined properties and applications, other fractions remain predominantly at the extraction and characterization stages.

**Table 1:** Structural components obtained from Amazonian bioresources and their technological applications.

Amazonian Resource	Structural Component	Processing/ Material	Key Technological Finding	Potential Application	Reference
*Croton lanjouwensis*; *Malvastrum tomentosum*; *Trema micrantha*	Lignocellulosic fibers	PBAT composites	Fibers increased PBAT elastic modulus by 48%, 70%, and 72%, respectively.	Biodegradable polymer composites	[60]
Patauá (*Oenocarpus bataua*) mesocarp	Lignocellulosic fiber	Raw and 5% NaOH-treated fibers	Alkali treatment exposed surface erosions, reduced fiber dimensions/density and increased crystallinity.	Biomaterial reinforcement	[68]
Curauá (*Ananas erectifolius*)	Lignocellulosic fiber	Treated-fiber biocomposites	Tensile strength was mainly governed by fiber fraction. A total of 25 wt.% treated fiber gave high tensile performance and treatment conditions-controlled adhesion.	Polymer biocomposites	[50]
Açaí, guaraná and tucumã residues	Lignocellulosic fibers	Injection-molded polypropylene biocomposites	Residues contained up to 50.9% cellulose. Composites showed distinct mechanical/thermal behavior, although tensile strength remained below neat PP.	Polymeric biocomposites	[65]
Jute and malva/ caesarweed	Cellulose nano-crystals	Delignification, bleaching and CNC extraction	Crystallinity increased from ~60% to ~85%. Extracted CNCs showed morphology and aspect ratio suitable for polymer-matrix reinforcement.	Nano-composite reinforcement	[69]
Açaí mesocarp residues	Cellulose nano-fibrils	Pretreatment + nano-fibrillation into films	Nano-fibrillation altered crystallinity, morphology and barrier behavior, supporting cellulose-film development from açaí residues.	Nano-structured films/ packaging	[63]
Paricá (*Schizolobium* *parahyba* var. *amazonicum*) waste	Cellulose nano-fibrils	Alkaline + bleaching treatments; CNF films	Bleaching reduced lignin from 26.1% to 6.8%. Chemical modification changed thermal, wetting and mechanical characteristics of CNF films.	Nano-structured cellulose films	[70]
Açaí residues	Cellulose nano-fibrils	Acid hydrolysis + ultrasonic processing	CNFs were 7.22–9.14 nm wide, with 75.78% crystallinity and high surface charge, demonstrating suitability for green nano-structured products.	Nano-structured materials	[71]
Arouma (*Ischnosiphon arouma*) stem residues	Cellulose/carboxymethyl cellulose	Cellulose extraction, CMC functionalization and film formation	Purified cellulose yielded water-soluble CMC. Films showed promising barrier and UV-blocking properties.	Bioplastics/food packaging	[72]
Amazon turmeric (*Curcuma longa*)	Starch	Single-matrix starch film	Starch produced a smooth biodegradable film with tensile resistance, elasticity and 65–92% antioxidant capacity by DPPH/ABTS.	Bioactive biodegradable films	[73]
Açaí seed flour + starch matrix	Starch-based biopolymer/lignocellulosic flour	Starch films with 0–15% açaí seed flour	5% flour gave the best distribution in the matrix. Incorporation modified packaging properties and added antioxidant functionality.	Active food packaging	[64]
Ucuuba cake + buriti pulp oil	Thermoplastic starch + lignocellulosic fiber	TPS biocomposite	Buriti oil reduced water absorption, improved processing flow and fiber adhesion, with a marginal strength increase but lower elongation.	Biodegradable biocomposites	[44]
Mandioca	Thermoplastic starch	Flat-sheet extrusion with plantain fiber/beeswax reinforcement	Reinforced TPS reached 5.99 MPa tensile strength and reduced WVTR to 366.6 g m^−2^ d^−1^ versus 0.48 MPa and 1486.6 g m^−2^ d^−1^ for control.	Flexible Packaging	[74]
Amazonian cocoa pod husk	RG-I-enriched pectin	High-pressure CO_2_ + water extraction	Pectic fractions reached 91.05% purity. Low methyl esterification and high acetylation favored emulsifier potential.	Food/pharmaceutical emulsifier; biomaterial feedstock	[61]
Açaí seeds	Crystalline mannan	Fractionation + NMR/XRD structural elucidation	Seeds contained a highly crystalline, predominantly linear β-(1→4)-mannan. The annose–galactose ratio was ~50:1.	Feedstock for mannose/mannan-oligosaccharides	[65]

### 4.2. Bioactive Compounds and Natural Extracts

Beyond structural components, Amazonian biodiversity provides a diverse source of bioactive compounds with technological potential. The studies selected in Table 2 primarily highlight phenolic compounds, carotenoids, pigments, and terpenes found in fruits, leaves, peels, and other plant fractions, mainly associated with antioxidant and antimicrobial properties and with potential applications in the food, cosmetic, and pharmaceutical sectors.

Phenolic compounds are among the most frequently reported bioactive compounds, including flavonoids, tannins, catechins, and procyanidins identified in various Amazonian resources. Açaí, inajá, babassu, murumuru, and the bark of several tree species have shown potential as sources of these compounds, often associated with high antioxidant activity. In the bark of *Byrsonima spicata*, *Tapirira guianensis*, and *Myrcia splendens*, for example, DPPH radical inhibition exceeded 92%, reaching values higher than those obtained for BHT under the evaluated conditions [75].

Carotenoids and natural pigments also stand out, particularly in Amazonian fruits. Peach palm (*Bactris gasipaes*) peels showed relevant carotenoid concentrations, while tucumã (*Astrocaryum vulgare*) was identified as a rich source of β-carotene [76]. Genipap (*Genipa americana*), in turn, enabled the production of a blue pigment containing genipin, geniposide, and other iridoids, broadening the potential use of these resources as natural sources of color and functionality [77].

Essential oils and terpenes represent another relevant group, with compounds such as linalool and β-caryophyllene primarily associated with antioxidant and antimicrobial activities. However, their composition can vary considerably. In *Aniba rosaeodora*, for example, linalool content in leaves ranged from 47.5% during the dry season to 74.4% during the rainy season [78]. This variation highlights the importance of considering raw material variability when standardizing Amazonian extracts for technological applications.

**Table 2:** Bioactive compounds and natural extracts obtained from Amazonian bioresources and their potential applications.

Amazonian Resource	Plant Fraction/ Material	Main Bioactive Compounds	Extraction/Processing Approach	Results	Potential Application	Reference
*Aniba* *rosaeodora* Ducke	Leaves and branches	Essential oil; linalool	Hydrodistillation; GC-MS; samples collected in wet and dry seasons	Linalool predominated in both fractions and varied seasonally, reaching 74.4% in leaves and 81.8% in branches during the wet season. The oils inhibited phytopathogenic fungi, particularly *Colletotrichum guaranicola.*	Natural antifungal agents; valorization of renewable aerial biomass	[78]
*Oenocarpus distichus* Mart. (bacaba-de-leque)	Fruit pulp and residual pulp after oil extraction	Vegetable oil; phenolic compounds; anthocyanins	Supercritical CO_2_ extraction (50–60 °C; different pressures)	Maximum oil yields were about 45%. After oil extraction, the residual pulp showed increased phenolic compounds, anthocyanins and antioxidant capacity, indicating that bioactive-rich fractions remained available for further recovery.	Nutraceutical ingredients; sequential biomass valorization	[12]
Açaí	Industrial processing residue	Polyphenols and antioxidant compounds	Microwave-assisted extraction; optimization of extraction conditions	Microwave-assisted processing improved recovery of polyphenolic compounds and antioxidant activity from açaí processing residues, supporting intensification of bioactive recovery from an abundant Amazonian byproduct.	Food, nutraceutical and natural antioxidant ingredients	[52]
*Maximiliana maripa* (Aubl.) Drude (inajá)	Oil-processing byproduct	Flavan-3-ols, catechins and procyanidins	Hydroethanolic extraction optimized by experimental design; LC-ESI-QTOF-MS	Extraction at approximately 70 °C using 50% ethanol favored recovery of phenolic antioxidants. Flavan-3-ols were the major phenolics and the extract showed antioxidant and anti-inflammatory-related responses.	Functional ingredients; nutraceutical, pharmaceutical and cosmetic applications	[79]
Açaí	Processing byproduct	Phenolic compounds	Pressurized liquid extraction; process optimization and techno-economic evaluation	Pressurized extraction enabled recovery of phenolic-rich fractions and was evaluated beyond laboratory yield through scale-up and economic considerations, providing evidence relevant to technological feasibility.	Natural antioxidant ingredients; scalable byproduct valorization	[18]
*Attalea speciosa* Mart. ex Spreng. (babassu)	Mesocarp	Phenolic compounds and flavonoids	Ultrasound-assisted hydroethanolic extraction	Extraction conditions, particularly the solid–liquid ratio, influenced phenolic recovery and antioxidant behavior, demonstrating the importance of process optimization for this Amazonian biomass.	Natural antioxidant extracts; food and nutraceutical ingredients	[80]
*Bactris gasipaes* Kunth (peach palm)	Fruit peels	Carotenoids, mainly β-carotene and γ-carotene isomers	Ultrasound-assisted extraction with ethanol compared with maceration, magnetic stirring and shaker extraction	Ultrasound-assisted extraction produced the highest carotenoid content (67 mg/100 g) and preserved the qualitative carotenoid profile, while replacing less desirable organic solvents with ethanol.	Natural colorants; carotenoid-rich food and functional ingredients	[81]
*Genipa* *americana* L. (genipap)	Fruit	Natural blue colorant; genipin, geniposide and other iridoids	Water-based ultrasound-assisted extraction; 70 °C, 325 W, 1:40 the solid–solvent ratio, 10 min	The optimized aqueous process recovered a blue extract containing genipin, geniposide and ten additional iridoids. The extract showed biological activity without cytotoxicity in the evaluated conditions.	Natural food colorants; pharmaceutical ingredients	[82]
*Pouteria* *macrophylla* (Lam.) Eyma (taturuba)	Plant material evaluated for bioactive extraction	Phenolic acids and flavonoids	Deep eutectic solvents (DES) and ultrasound-assisted extraction compared with ethanol/conventional approaches	Solvent composition and extraction technology selectively affected the recovery of different phenolic classes, illustrating the potential of DES as alternative media for obtaining Amazonian bioactives.	Green extraction; functional and nutraceutical ingredients	[83]
*Byrsonima* *Spicata; Croton matourensis*; *Myrcia* *splendens*; *Tapirira* *guianensis*; *Vismia* *guianensis*	Tree barks collected in Pará, Brazil	Condensed tannins, flavonoids and phenolic acids	Extraction followed by colorimetric assays and HPLC	*M. splendens*, *V. guianensis* and *B. spicata* contained 841.8, 544.5 and 503.6 mg catechin equivalents/g extract of condensed tannins, respectively. Several extracts showed >92% DPPH inhibition and outperformed BHT under the tested conditions.	Natural antioxidants; possible alternatives to synthetic phenolic antioxidants	[75]
*Calycolpus goetheanus* (O. Berg)	Plant material/volatile fraction	Terpenes and fatty alcohols	Supercritical CO_2_ extraction at 35–45 °C and 150–350 bar; comparison with hydrodistillation	The highest extraction yield was 2.11% at 45 °C/350 bar. Supercritical extracts showed composition-dependent antioxidant activity and generally superior performance to hydrodistillation extracts.	Pharmaceutical, cosmetic and nutraceutical extracts	[84]
*Copaifera* *reticulata* Ducke	Leaves	β-Caryophyllene-rich extract	Supercritical CO_2_ extraction	Supercritical extraction concentrated β-caryophyllene in leaf extracts, demonstrating the potential of renewable aerial biomass as an alternative source of a high-value terpene rather than relying exclusively on trunk oleoresin.	Cosmetic, pharmaceutical and natural-product ingredients	[85]
*Astrocaryum murumuru* Mart.	Residual biomass from oilseed processing	Phenolic compounds, flavonoids and tannins	Ultrasound-assisted extraction with ethanol; central composite rotational design	Optimal conditions (60% ethanol, 2.5% m/v solid–liquid ratio, 26 min) yielded 9.92 mg GAE/g total phenolics, 0.38 mg QE/g flavonoids and 4.03 mg TA/g tannins; extracts also showed antioxidant activity.	Natural food additives; antioxidant extracts; sustainable byproduct valorization	[84]
*Astrocaryum vulgare* Mart. (tucumã)	Fruit pulp	β-Carotene-rich oil; oleic acid; phenolics and flavonoids	Compressed propane extraction compared with Soxhlet	Compressed propane extraction reached 33.9 wt.% yield and up to 91.4% extraction efficiency. The resulting oil contained about 21-fold more β-carotene than oils obtained by Soxhlet.	Food, cosmetic and pharmaceutical oils; green extraction	[77]
*Bactris gasipaes* Mart. (peach palm)	Fruit peels	Carotenoids	Supercritical CO_2_ extraction at different pressures and densities	The highest carotenoid concentration (2446.9 µg/g) was obtained at 40 °C and 400 bar. Extracts also exhibited antioxidant capacity and 81–83% inhibition against selected microorganisms.	Carotenoid-rich natural extracts; food additives and antimicrobial ingredients	[86]

Another recurring aspect is the valorization of peels, residues, and remaining processing fractions as sources of bioactive compounds. In bacaba-de-leque, for example, the residual pulp after oil extraction retained phenolic compounds, anthocyanins, and antioxidant capacity [12,14]. This sequential valorization brings these production chains closer to the biorefinery concept, in which different components from the same biomass can be recovered and directed toward higher-value products, thereby maximizing the utilization of already harvested Amazonian resources.

## 5. Micro and Nanotechnology Approaches

Micro- and nano-structuring strategies have been increasingly explored to preserve bioactive compounds [29,87,88] for food and pharmaceutical applications; however, few studies have explored Amazonian bioresources to promote their stability and incorporation into technologically relevant systems, as summarized in Table 3.

Available studies indicate that stabilization is not an intrinsic consequence of encapsulation. Instead, these systems’ performance depends on the interplay among processing conditions, carrier composition, and the physicochemical characteristics of the incorporated compounds. This relationship is particularly evident in spray-dried systems [89,90,91,92], where wall materials simultaneously influence feed properties, particle formation, powder recovery, and the stability of bioactive compounds. During micro-encapsulation of cupuaçu (*Theobroma grandiflorum*) and açaí (*Euterpe precatoria*) bioactive compounds, increasing maltodextrin concentration did not significantly improve process yield and, at higher proportions, lower yields of approximately 60–62% were observed [89]. Although maltodextrin can increase the glass transition temperature (Tg) and thereby reduce stickiness, excessive concentrations may increase feed viscosity, impair droplet atomization and drying, and consequently favor particle deposition on the drying chamber walls. At 9.6%, maltodextrin partially preserved phenolics and flavonoids [89]. In contrast, microencapsulation of açaí extract using a combination of gum Arabic (GA) and maltodextrin (MD) demonstrated the advantage of combining wall materials with complementary physicochemical properties. GA increased Tg and stabilized the feed system, whereas MD’s comparatively low viscosity favored atomization. At a 50:50 ratio, the system retained phenolic compounds while maintaining adequate drying efficiency. Thus, optimization of the encapsulating matrix appears to depend less on increasing the concentration of a single wall material than on achieving an appropriate balance among Tg, viscosity, atomization behavior, and interaction with the encapsulated compounds.

Additionally, processing temperature represents another critical variable because conditions that favor efficient particle formation affect bioactive compounds’ stability. In studies with cupuaçu and açaí, higher drying temperatures favored retention of total phenolics and flavonoids, attributed to rapid water evaporation and shorter drying times that limited oxygen exposure and oxidative reactions [89]. Nevertheless, this response was compound-dependent, as higher temperatures negatively affected more thermolabile compounds, particularly anthocyanins and ascorbic acid. A similar finding was reported for spray-dried açaí, where inlet-air temperature had to be balanced against the potential thermal degradation of the encapsulated bioactive compounds [90]. These observations show that high drying temperatures should not necessarily be interpreted as detrimental to all bioactive compounds, since rapid drying may favor retaining some of them, with losses of their thermolabile constituents. Consequently, the protective capacity of the encapsulating matrix has practical and thermodynamic limits that modifying only wall-material composition cannot overcome. Optimization should therefore consider the stability of individual classes of bioactive compounds rather than relying exclusively on global responses such as total phenolic content or antioxidant capacity.

At the nano-scale, the relationship between the bioactive compounds and the carrier becomes particularly strategic, as the incorporated compounds may influence the physicochemical organization of the resulting biomaterial. For instance, patawa (*Oenocarpus bataua*) fruit extract encapsulated in acetylated cashew gum/chitosan nano-particles modified the structural organization of the polymeric system, as evidenced by the appearance of a characteristic diffraction peak, and changes in enthalpy affected the thermal behavior and organization (crystallinity) of the obtained nano-particles [95]. Similarly, the nano-encapsulation behavior of Amazonian *Piper alatipetiolatum* essential oil and zerumbone in PEG-PCL nano-particles was associated with differences in their chemical characteristics. Zerumbone’s defined structure and marked hydrophobicity were considered favorable for its interaction with the hydrophobic PCL core, whereas the essential oil’s greater chemical complexity, comprising terpenes with different partitioning behaviors, was associated with lower retention within the polymeric matrix [91]. Hence, these findings emphasize that nano-encapsulation involves molecular interactions between the carrier and the incorporated bioactive compounds, which can determine both bioactive retention and the structural organization of the resulting nano-structures.

A distinct nano-scale application of Amazonian bioresources is the green synthesis of metallic nano-particles, in which plant extracts act primarily as reducing and stabilizing agents rather than as bioactive compounds carried by a nano-structured matrix. In açaí-mediated synthesis, anthocyanins were proposed to act as capping agents, while the aqueous extract exhibited both reducing and stabilizing capacities, enabling the formation of small spherical AgNPs [96]. Similar approaches using guaraná and tucumã further demonstrate the potential of Amazonian extracts for green nano-particle synthesis, although nano-particle characteristics strongly depend on the bioresource and synthesis conditions. Guaraná-mediated AgNPs exhibited colloidal stability over two years, whereas temperature exerted contrasting effects on açaí- and tucumã-mediated synthesis. Higher temperatures favored the formation of smaller, more homogeneous açaí-mediated AgNPs, while increasing temperature enhanced the silver-reducing capacity of tucumã extract; however, temperatures above 40 °C impaired stabilization, promoting aggregation and greater polydispersity [97]. These findings reveal an important distinction between nano-particle formation and nano-particle stabilization. These conditions, which favor metal-ion reduction, do not necessarily provide optimal colloidal stability.

For carrier-based nano-structured systems, encapsulation efficiency alone does not adequately describe the technological or biological performance of these nano-composites. Despite moderate encapsulation efficiencies (33%; Table 3), the *Piper* or zerumbone-PEG-PCL formulations maintained biologically relevant effects and significantly reduced Zika virus in adsorption, replication, and inactivation stages [91]. Likewise, nano-encapsulation of patawa extract markedly enhanced its biological performance, increasing antioxidant responses and producing antibacterial activity against *Escherichia coli*, *Salmonella enteritidis*, and *Staphylococcus aureus* [95]. The antibacterial effect was associated with nano-encapsulation’s ability to concentrate phenolic compounds within the microenvironment surrounding bacterial membranes, where extensive cellular damage and leakage of intracellular components were observed. Therefore, the benefits of nano-encapsulation do not depend solely on successfully entrapping bioactive material. Particle size and dispersity, colloidal stability, bioactive loading, and preservation or enhancement of biological activity determine whether a nano-composite carrying Amazonian bioactive compounds provides a meaningful functional performance.

Despite these promising technological and biological properties, safety must be considered when evaluating the potential applications of Amazonian-derived micro- and nano-systems. In particular, reported cytotoxicity of guaraná-mediated nano-particles toward non-cancerous cells indicates that biological activity does not necessarily imply selective or safe performance [93]. Furthermore, findings in *C. elegans* should be interpreted primarily in the context of environmental and ecotoxicological safety rather than as evidence supporting pharmaceutical use. Therefore, future studies should integrate technological performance with cytotoxicity, biocompatibility, and environmental safety assessments before proposing specific applications for these nano-structured systems.

## 6. Technological Properties and Applications of Biomaterials Incorporating Amazonian Bioresources

The chemical and structural diversity of Amazonian bioresources enables their incorporation into biomaterials designed for several technological purposes, including food, cosmetic, pharmaceutical, and biotechnology industries. Their role across these sectors ranges from bioactive ingredients that provide reinforcement, antioxidant, antimicrobial, or other biological properties to targeted biomaterials for specific applications. Current applications are concentrated particularly in food and packaging, pharmaceutical and biomedical, cosmetic and dermocosmetic, and structural and engineering fields.

Amazonian fibers, extracts, and oils have been incorporated into polymeric matrices for food packaging purposes to introduce antioxidant or antimicrobial activity, although their presence can simultaneously alter mechanical, barrier, thermal, and water-related properties, allowing alternatives to conventional food packaging that still can protect the food during transport and commercialization while extending the products’ shelf life [98,99,100]. Açaí oil incorporated into fish-gelatin edible coatings modified the structural organization of the matrix and contributed to the preservation of tomato quality during storage, reducing mass loss and maintaining greater firmness [98], whereas buriti (*Mauritia flexuosa*) oil incorporated into polysaccharide-based films increased tensile strength and elastic modulus accompanied by decreased elongation at break in the films [101]. These applications show that Amazonian bioresources may contribute to food packaging beyond the direct biological activity of their phytochemicals, since interactions between the incorporated compounds and the polymeric network can determine the mechanical and mass-transfer behavior of the resulting material.

Regarding pharmaceutical applications, açaí-mediated silver nano-particles (AgNP) exhibited antimicrobial activity with minimal toxicity in *C. elegans* at environmentally relevant concentrations [96], whereas guaraná-derived nano-particles showed cytotoxic and larvicidal effects [94]. *Aniba canelilla* essential oil has shown potential for pharmaceutical and cosmetic applications when incorporated into nano-emulsions thickened with hydroxyethylcellulose or chitosan hydrogels. The formulations exhibited nano-metric droplet sizes (~150 nm), controlled release, and enhanced retention of the major oil constituents in the epidermis and dermis. Notably, the hydroxyethylcellulose-based system preserved the oil’s anti-inflammatory activity, reducing edema and inflammatory markers and supporting nano-structured polymeric matrices as promising platforms for topical delivery of Amazonian bioactives [3,25,102]. These contrasting biological responses highlight the distinct functional profiles of Amazonian-mediated nano-materials and, consequently, their potential suitability for different applications.

## 7. Sustainability and Circular Economy Perspectives

Sustainability in the use of Amazonian resources should not be understood exclusively in terms of valorizing residues generated by production chains [103]. In a biome characterized by high biodiversity and simultaneously subjected to multiple pressures from natural resource exploitation and land-use conversion, the economic valorization of products derived from standing forests is a particularly relevant perspective [104]. In this context, the Amazonian bioeconomy has been proposed as a model that generates value from biodiversity while promoting the maintenance of a living, functional forest, in contrast to economic systems that depend on forest clearing or its replacement with simplified production systems [103,105].

This perspective broadens the possibilities for utilizing plant resources beyond, for example, timber exploitation. Fruits, seeds, oils, resins, fibers, leaves, and other plant components can serve as sources of bioactive compounds and materials of technological interest without necessarily removing the plant [103]. These resources, often classified as non-timber forest products, can be integrated into higher-value chains across the food, cosmetic, pharmaceutical, and biomaterials sectors. Thus, economic value is no longer tied exclusively to extracting forest biomass but also to maintaining the organisms and ecosystems that renew these resources [104,105].

However, these products are not inherently sustainable. The intensity and frequency of harvesting, the plant part exploited, and each species’ regenerative capacity can determine the ecological impacts of these activities. Excessive harvesting of leaves, bark, or other plant structures, for example, may compromise physiological processes, plant growth, and reproduction. Therefore, the contribution of these resources to forest conservation depends on adopting appropriate management practices and thoroughly understanding each species’ ecological characteristics [106,107].

The circular economy adds a second dimension by maximizing the use of biomass that has already entered production chains. These principles are already applied, for example, in the açaí (*Euterpe oleracea*) value chain, where seeds can account for up to 80–95% of the fruit mass and are generated in large quantities during pulp production [108]. Lima et al. [109] roasted and ground açaí seeds to produce a naturally caffeine-free coffee alternative. The extract prepared at 100 °C contained 21.78 mg GAE/g of phenolic compounds and exhibited an antioxidant capacity of 183.33 µmol TE/g, as determined by the FRAP assay, demonstrating that an abundant residual fraction can be converted into a functional food product. Commercial samples of this beverage also showed phenolic compounds and antioxidant activity after simulated gastrointestinal digestion, further supporting the potential valorization of açaí seeds for food applications [108].

The valorization of these materials is also not limited to food applications. For example, the solid co-product remaining after oil extraction from cupuaçu (*Theobroma grandiflorum*) seeds still contained 16.9 ± 1.8 mg/g of polyphenols, as well as epicatechin and glycosylated quercetin, indicating that sequential processing steps may enable the additional recovery of compounds of interest from biomass that has already undergone industrial processing [110].

Amazonian agro-industrial residues have also been used to develop materials for environmental applications. Açaí seeds, Brazil nut shells, oil palm shells, and babassu residues have been evaluated as precursors for activated carbon production, demonstrating the potential to convert these residual biomasses into materials with higher technological value [111]. These examples show that circularity can occur at different levels within the same value chain, from directly transforming residues into new food products to using them as sources of bioactive compounds or as raw materials for functional materials, thereby increasing the value derived from already harvested Amazonian biomass.

In this context, sustainability and circularity can complement each other in valorizing Amazonian biodiversity. While the sustainable management of renewable resources enables economic value generation through forest conservation, the subsequent utilization of residues and co-products seeks to maximize the value derived from already harvested biomass and contribute to the transition toward a circular model. However, demonstrating the technical potential for residue valorization does not necessarily ensure the overall environmental sustainability or economic viability of these pathways. Quantitative tools, such as life cycle assessment (LCA), techno-economic analysis (TEA), and process integration, are therefore important for comparing alternative valorization routes and identifying environmental and economic trade-offs. Integrated approaches applied to food-waste biorefineries have combined environmental and economic assessments and, more recently, multi-objective optimization considering indicators such as global warming potential (GWP), net present value (NPV), and employment generation [106,108,110]. Applying similar frameworks to Amazonian bioresources could support comparing and prioritizing different product routes from residues such as açaí seeds and cupuaçu processing byproducts, considering process yields, energy and material requirements, environmental impacts, economic performance, and opportunities for process integration.

These quantitative assessments could also provide a measurable connection between the circular biorefinery approach illustrated in Figure 2 and the Sustainable Development Goals (SDGs). Integrated environmental and techno-economic assessments of biorefineries can support evaluation of SDG 7 (Affordable and Clean Energy), SDG 12 (Responsible Consumption and Production), and SDG 13 (Climate Action) through indicators related to energy use and recovery, resource efficiency and waste valorization, and life-cycle environmental impacts, respectively [111,112]. Economic and social indicators, including profitability and employment generation, can further complement this assessment by addressing the socioeconomic dimension of biorefinery development [113]. Therefore, integrating LCA, TEA, and socioeconomic indicators could provide a quantitative basis for assessing whether valorization pathways proposed for Amazonian residues effectively contribute to circular bioeconomy and sustainable development objectives.

Beyond ecological considerations, the sustainable use of Amazonian biodiversity also involves social and regulatory dimensions, particularly the participation of Indigenous Peoples and traditional communities and the fair and equitable sharing of benefits arising from the use of genetic resources and associated traditional knowledge. In this context, the Nagoya Protocol and Brazilian Law No. 13,123/2015 [114] provide relevant frameworks for access to genetic resources and associated traditional knowledge and for benefit sharing, reinforcing the need to integrate technological and economic development with the responsible and equitable use of Amazonian biodiversity.

## 8. Landscape, Challenges, and Future Perspectives

The current landscape of Amazonian-derived biomaterials reflects a shift from conventional biodiversity exploitation to integration into more complex, reinforced biomaterial platforms loaded with bioactive compounds. Plant extracts, oils, polysaccharides, lignocellulosic residues, and natural fibers have been incorporated into micro- and nano-structures, films, food, food packaging, and reinforced composites, supporting applications ranging from food preservation and bioactive delivery to cosmetics and structural materials [108,109,115,116,117]. Particularly relevant is the potential to exploit different fractions of the same biomass for distinct technological purposes, including recovering high-value compounds followed by producing structural materials.

However, the technological maturity of these applications remains heterogeneous. While fibers, cellulose, and starch have been incorporated into composites, films, and packaging systems with measurable mechanical and barrier properties, other Amazonian fractions remain largely at the extraction, characterization, or proof-of-concept stage, as shown in Table 4. A similar pattern is observed for bioactive compounds, for which antioxidant or antimicrobial activity is frequently demonstrated, but their performance after incorporation into final products is evaluated less consistently. Therefore, the diversity of Amazonian bioresources identified in the literature has not yet translated into an equivalent diversity of technologically validated products, revealing an important gap between resource characterization and application-oriented development.

Nevertheless, the existing literature largely comprises laboratory-scale studies, making it difficult to assess progress toward technologically and economically viable applications. Moreover, the intrinsic variability of biological resources due to species, seasonality, ripeness, and processing conditions may affect both bioactive composition and material performance, highlighting the need for greater raw-material characterization and standardization. Additionally, future research should therefore prioritize application-specific validation, long-term stability, toxicological assessment, and process scalability. Regarding the nano-technology approach, future studies should move beyond demonstrating particle formation or reporting encapsulation efficiency alone and establish whether structuring effectively improves bioactive retention, physicochemical stability, and biological performance relative to the corresponding non-encapsulated bioresource under relevant processing and storage conditions.

## Figures and Tables

**Table 3:** Micro- and nano-systems carrying Amazonian bioactive compounds for the development of biomaterials and their technological and biological potentials.

Amazonian Bioresource	Primary Materials	Approach	Composite Profile and Application	Results	Reference
Cupuaçu; açaí	Cupuaçu and açaí pulps (1:1 *w*/*w*) combined with Cupuaçu seeds aqueous extract (0–15%)	Spray drying (10% total solids) using peristaltic pump with feed rate of 4.5 mL/min, 0.06 MPa, 0.7 mm nozzle diameter at 165 °C, 9.6% maltodextrin as wall material and 11.3% seed extract was the optimized condition	Microcapsules with roughly spherical morphology (average particle size 8.68 μm). The authors did not propose any application.	Yield of 76.6%, solubility of 87%. Wall material concentration did not determine yield but negatively affected TPC, TFC, ABTS, and FRAP per gram of powdered microcapsules. Higher drying temperatures positively affected TPC and TFC and did not affect FRAP, but negatively affected anthocyanins and ascorbic acid contents. Cupuaçu seed extract provided phenolics and flavonoids, and the spray drying technique partially preserved the bioactive compounds	[89]
Açaí	Açaí pulp ethanolic extract; Arabic gum and maltodextrin	Spray drying (8% total solids) with Arabic gum and maltodextrin (1:1) as wall material and 70% ethanolic açaí extract. The feed rate was 10 mL/min, 0.06 MPa, 1.5 mm nozzle diameter at 135 °C	Microcapsules with roughly spherical morphology (size ranging from 1.19 to 24.94 μm). The authors did not propose any application.	Arabic gum improved the process yield. The obtained biomaterial is nonhygroscopic, contributing to the shelf life of the microcapsules powder. TPC reached 2803.29 mg GAE/100 g, with a thermal and oxidative protective role of the encapsulation matrix	[90]
*Piper* *alatipetiolatum* Yunk.	Piperaceae leaves essential oil (10% *w*/*w*); Zingiberaceae rhizome (*Zingiber zerumbet* L.) (10% *w*/*w*); PCL (4.5 mmol); PEG (0.125 mmol). PEG-PCL were copolymers (5 mg/mL)	Nano-precipitation under stirring for 15 min followed by filtration through 0.22 μm membrane	Nano-particles (average particle size of 62.84 nm ± 2 (leaves essential oil) and 74.86 ± 3 (zerumbone)) with a well-defined core–shell arrangement, core. The authors refer to potential application as a platform for Zika virus targeted interventions.	The encapsulation efficiency was 33% (leaves) and 37% (rhizome). Sesquiterpenes were the majority compounds in the essential oil. All nano-formulations exhibited significant anti-Zika virus effects (*p* < 0.001) in vitro. Zerumbone loaded nano-particles displayed the most consistent profile across adsorption, replication, and inactivation stages of Zika virus	[91]
Buriti	Buriti crude oil; soy protein isolate (5–15% *w*/*w*) and maltodextrin (5–15% *w*/*w*) as wall material	Spray drying O/W emulsions (10% buriti oil and 5.45% soy protein isolated combined with 14.55% maltodextrin). The feed rate was 3 mL/min, 3–5 bar, 0.7 mm nozzle, at 180 °C was the optimized condition	Heterogeneous microcapsules with spherical morphology (particle size from 2.75 to 4.18 μm). O/W emulsions. The authors suggest the application in baked goods, dairy, and dairy-alternative products, as well as supplements.	Yield of 94.95 ± 1.10%, solubility 94.09 ± 1.23%. The microencapsulation contributed to the thermal stabilization of the buriti oil. Soy protein isolate ocasionated high yields and oil encapsulation efficiency, protected against carotenoid oxidation.	[92]
Guaraná (*Paullinia cupana*)	Guaraná leaves aqueous extract (2 mg/mL) combined with aqueous solution of AgNO_3_ at 2 mM (340 μg/mL) at final volume of 50 mL	Green synthesis of silver nano-particles (AgNP), incubated in water bath at 70 °C during 180 min	Nano-particles (dry diameter size from 39.33 nm to 42.43 nm). The morphology varied between spherical, quasi-spherical, hexagonal, prismatic, among others. The authors refer to potential application in health, industrial and environmental sectors.	The silver content varied between 18.03 and 26.5%, and the system had colloidal stability over two years of evaluated storage. For DPPH and ABTS antioxidant activity, the nano-particles had statistical lower values compared to the Guaraná extract in all the tested concentration. The nano-particles showed cytotoxic effect against cancerous and non-cancerous cell lines (from 400 to 2000 μg/mL) and larvicidal effect against *Aedes aegypt.* The Guarana extract isolated was not cytotoxic in all concentrations.	[93]
Tucumã (*Astrocaryum vulgare*); jambu (*Acmella oleracea*)	Tucumã butter (225 mg); jambu oil (225 mg); curcumin (*Curcuma longa*) (22.5 mg); surfactant (450 mg); Natural Deep Eutectic Solvent and water (1:1)	Nano-structured lipid carrier where the lipid phase was melted at 80 °C, the aqueous phase was added, and solidification were performed in ice bath for 1 min	Nano-structures (average particle size 221.03 ± 32.72 nm). The authors refer to highly effective application in nano-carriers, pharmaceutical or cosmetic formulations for sensitive skin, and barrier repair dermocosmetics.	The encapsulation efficiency of curcumin was 81.39%. Natural Deep Eutectic Solvent improved stability of bioactive compounds in the formulation. The obtained structures promoted fibroblast adhesion and proliferation and modulated inflammatory responses, including the expression of the anti-inflammatory cytokine IL-4.	[94]
Patawa (*Oenocarpus bataua*)	Patawa fruti ethanolic extract (1.65 mL), acetylated cashew gum polysaccharide (75 mg) and chitosan (25 mg) in a final volume of 50 mL	Nano-precipitation under high shear homogenization at 22 rpm for 15 min	Nano-particles (average particle size of 234.7 ± 10.3 nm). The authors refer to potential application in active food packaging.	The encapsulation efficiency was 40.1%. The main bioactive compounds were epicatechin (38.20%), naringenin (14.39%) and rutin (12.47%). Patawa extract improved the chemical and thermal behavior of the nano-particles. The nano-encapsulation can efficiently improve the antioxidant activity of active ingredients (45-fold in DPPH, 20-fold in ABTS and 5-fold in FRAP) and antibacterial activity against *Escherichia coli*, *Salmonella enteritidis*, and *Staphylococcus aureus*	[95]
Açaí	Açaí pulp aqueous extract (100 mg/mL); AgNO_3_ (10 mg/mL)	Biosynthesis of AgNP performed at 45 or 70 °C, for 90 min at 36 rpm in a water bath	Spherical nano-particles (hydrodynamic diameter of 150 nm when synthesized at 70 °C and 180 nm at 45 °C)	At higher temperatures, more homogeneous nano-particles were obtained, but both temperatures achieved moderated stability with negatively charged surface. The nano-particles had antimicrobial activity, with minimal toxicity in *Caenorhabditis elegans*. These findings indicate that açaí-mediated biosynthesis is a promising approach for producing antimicrobial AgNP with low toxicity at environmentally relevant concentrations.	[96]
Tucumã (*Astrocaryum aculeatum*)	Tucumã pulp extract (1; 1.5 and 2 g/100 mL concentration) (5 mL); AgNO_3_ (0.001 mmol/L; 50 mL)	The colloidal dispersions were prepared at pH 5.0; 7.0 and 9.0 under constant agitation in vessels at 30, 40 and 50 °C for 24 h	Spherical nano-particles (average particle size from 18.8 ± 1.0 to 77.0 ± 3.8).	The results suggest that higher temperatures enhanced the silver-reducing capacity of tucumã extract without degrading the biomolecules involved in the reduction process. Temperatures above 40 °C impaired silver nano-structure stabilization, promoting aggregation and greater polydispersity in hydrodynamic diameter. The best formulation was at Tucumã concentration of 1.5 g/100 mL at 40 °C and pH 9.0	[97]

**Table 4:** Comparative overview of the development stage and research gaps of Amazonian bioresources and derived materials.

Resource/Material	Evidence Across Studies	Development Stage	Main Gap	References
Structural biomaterials (fibers, cellulose/nano-cellulose, and starch)	Production of composites, films, and reinforced materials with measurable mechanical, thermal, and/or barrier properties.	Material/application validation	Standardization and validation beyond laboratory scale.	[44,60,62,63,64,65,71,72,73,74]
Bioactive compounds and extracts	Extraction optimization and demonstration of antioxidant, antimicrobial, and other biological activities.	Extraction/functional validation	Validation after incorporation into final products and assessment of compositional variability.	[12,45,52]
Micro- and nano-structured systems	Encapsulation, nano-emulsions, and nano-particles with physicochemical and biological characterization.	Proof-of-concept/application validation	Stability, safety, reproducibility, and comparison with non-structured systems.	[89,90,91,92,93]
Integrated biomass valorization	Sequential recovery of bioactive and structural fractions from residues and processing byproducts.	Process-level demonstration	Limited integrated scale-up, LCA, TEA, and process-integration assessments.	[56,106,108]

## Data Availability

No new data were created or analyzed in this study. Data sharing is not applicable to this article.

## Abbreviations

The following abbreviations were used in this manuscript:

ABTS	2,2′-Azino-bis(3-ethylbenzothiazoline-6-sulfonic acid)
AgNPs	Silver nano-particles
AgNO_3_	Silver nitrate
BHT	Butylated hydroxytoluene
CMC	Carboxymethyl cellulose
CNCs	Cellulose nano-crystals
CNFs	Cellulose nano-fibrils
DPPH	2,2-Diphenyl-1-picrylhydrazyl
FRAP	Ferric reducing antioxidant power
FTIR	Fourier-transform infrared spectroscopy
GA	Gum Arabic
GAE	Gallic acid equivalents
GC–MS	Gas chromatography–mass spectrometry
GWP	Global warming potential
HPLC	High-performance liquid chromatography
IL-4	Interleukin-4
LCA	Life cycle assessment
MD	Maltodextrin
NPV	Net present value
O/W	Oil-in-water
PBAT	Poly(butylene adipate-co-terephthalate)
PCL	Poly(ε-caprolactone)
PEG	Polyethylene glycol
PP	Polypropylene
PRISMA	Preferred Reporting Items for Systematic Reviews and Meta-Analyses
RG-I	Rhamnogalacturonan I
SDGs	Sustainable Development Goals
SEM	Scanning electron microscopy
TEA	Techno-economic analysis
TFC	Total flavonoid content
TPC	Total phenolic content
TPS	Thermoplastic starch
WVTR	Water vapor transmission rate
